# Effects of Mental Fatigue on *Small-World* Brain Functional Network Organization

**DOI:** 10.1155/2019/1716074

**Published:** 2019-12-06

**Authors:** Gang Li, Youdong Luo, Zhengru Zhang, Yanting Xu, Weidong Jiao, Yonghua Jiang, Shan Huang, Chengwu Wang

**Affiliations:** College of Engineering, Zhejiang Normal University, Jinhua 321004, China

## Abstract

Brain functional network has been widely applied to investigate brain function changes among different conditions and proved to be a *small-world*-like network. But seldom researches explore the effects of mental fatigue on the *small-world* brain functional network organization. In the present study, 20 healthy individuals were included to do a consecutive mental arithmetic task to induce mental fatigue, and scalp electroencephalogram (EEG) signals were recorded before and after the task. Correlations between all pairs of EEG channels were determined by mutual information (MI). The resulting adjacency matrices were converted into brain functional networks by applying a threshold, and then, the clustering coefficient (*C*), characteristic path length (*L*), and corresponding *small-world* feature were calculated. Through performing analysis of variance (ANOVA) on the mean MI for every EEG rhythm, only the data of *α*1 rhythm during the task state were emerged for the further explorations of mental fatigue. For a wide range of thresholds, *C* increased and *L* and *small-world* feature decreased with the deepening mental fatigue. The pattern of the *small-world* characteristic still existed when computed with a constant degree. Our present findings indicated that more functional connectivities were activated at the mental fatigue stage for efficient information transmission and processing, and mental fatigue can be characterized by a reduced *small-world* network characteristic. Our results provide a new perspective to understand the neural mechanisms of mental fatigue based on complex network theories.

## 1. Introduction

Mental fatigue refers to a status that decreased mental alertness and focus and worsening performances [1, 2]. And it is often caused by prolonged periods of cognitive activities. Mental fatigue has become one of the most common subhealthy states in modern society and affects nearly all aspects of cognitive functioning in humans, especially in fatal traffic accidents [3–5]. Concerning the effects of mental fatigue on our daily life, it is important to explore the neurocognitive mechanisms of mental fatigue based on complex network theories.

Brain functional network, as one type of the complex networks in statistical physics, is a demonstration of the temporal correlations among the different brain regions in the course of nervous activities [6]. It has become one of the most widely used methods to investigate neurodynamics of cognitive functions [7–9], which are especially sensitive to mental fatigue [10, 11]. Commonly explored neuroimaging techniques of brain functional networks are mainly on the basis of electroencephalogram (EEG) data [12], because EEG has the advantages of high temporal resolution, low costs, and easy operation.

Recent advances in the development of quantitative EEG analysis have allowed the exploration of functional interactions across the cerebral cortex during mental fatigue [13]. With increasing levels of mental fatigue, functional connectivity patterns dynamically changed to meet the balanced functional integration and segregation between different cortical areas [13, 14], and the obtained network parameters, such as degree, *C*, and *L*, also significantly changed at successive stages of the experiment [15]. It has been widely proved that brain functional networks have the *small-world* property since Kar and Routray [15] revealed the *small-world* characteristic of most real networks in 1998. A *small-world* network, with a high *C* and a short *L* [16], has been proposed as a sign of “optimal organization” during specific functions [16–18]. The *small-world* feature is also obtained in brain structural (anatomical) networks revealed by cortical thickness [19]. The values of *small-world* vary along with the change of brain functions [20, 21]. However, the influences of mental fatigue on the *small-world* brain functional network structures are still equivocal.

In the current study, we attempted to investigate the effects of mental fatigue on brain functional network organization indexed by the *small-world*. To achieve this goal, firstly, we induced mental fatigue among the participants by a mental arithmetic math task and validated mental fatigue with a power algorithm. Previous researchers have reported that the power of low wave (*θ* and *α* activities) increases and the power of fast wave (*β* activity) decreases during mental fatigue [22, 23]. So, we chose the spectral power ratio of (*θ* + *α*)/*β* for mental fatigue detection, which has been proved to be the best indicator on account of its highest classification capability [24]. Secondly, we formed the full-weighted adjacency matrix (AM) with multichannel EEG data. Mutual information (MI) was applied to determine the functional connectivity among all pairwise combinations of EEG channels; MI can not only characterize the functional interaction from the point of information transformation between different brain regions based on information theory but also quantify the comprehensive information both signal phase and signal amplitude [9]. Then, AM was converted into a brain functional network by applying a threshold for further network analysis (*C*, *L*, and *small-world*) underlying the impacts of mental fatigue.

## 2. Materials and Methods

### 2.1. Participants

Twenty healthy male volunteers (females were excluded to eliminate the effects of sex difference on the results) of engineering graduate students from Shandong University were recruited. Their average age was 24.5 ± 1.5 years, and their body mass index was 20.7 ± 1.8 kg/m^2^. Every participant should be right-handed and should have a regular living habit, normal or rectified normal eyesight, and no brain diseases. Every subject was required to do as follows: not staying up late at night and not drinking alcohol and drugs in one week preceding the EEG recording, not smoking and drinking coffee and tea in 8 hours before EEG data acquisition, and washing their hair in 2 hours before the experiment. Each subject was informed the experimental procedures, and informed consent was gathered from every participant. The local Ethics Committee have approved this study. Every subject got some monetary reimbursement to motivate their better cooperation during the whole experiment.

### 2.2. EEG Data Recording

Scalp EEG data were collected with an apparatus (SYMTOP NT9200) at the following nineteen electrodes in 10-20 systems: Fp1, Fp2, F3, F4, C3, C4, P3, P4, O1, O2, F7, F8, T3, T4, T5, T6, Fz, Cz, and Pz (A1 and A2 were chosen as the reference electrodes; Fpz was selected to be the grounding electrode). Electrode impedance was controlled under 5000 *Ω*. Sample frequency was 1000 Hz. For the sake of inducing mental fatigue, all participants were asked to do a mental arithmetic task with 200 different problems for 100 minutes. The mental arithmetic problem is that a random double-digit (between 60 and 90) plus another random double-digit (between 60 and 90) and then multiplied by a random single digit (between 6 and 9). Each math problem was designed to be completed in 30 seconds determined by preceding pretests.

**Figure 1:**
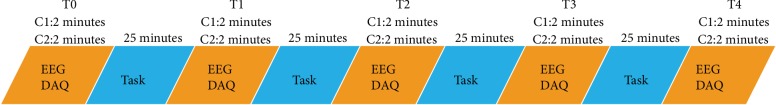
EEG data acquisition (EEG DAQ) procedures.

As shown in Figure 1, the whole mental arithmetic task was equally divided into 4 tasks, and EEG was collected before and after each task. Therefore, there were 5 times of EEG data acquisition named as T0, T1, T2, T3, and T4 for the whole task. Besides, two conditions were considered for each data acquisition: resting state (C1) and task state (C2), and 2 minutes of the EEG signals was collected for each condition. The resting state means closing the eyes, being awake and relaxed, and subjects were required to concentrate their attention on the breath avoiding thinking about anything, whereas the task state refers to keeping the body still and doing a mental arithmetic math problem, a three-digit subtracts a single digit continuously (keep same for each data collections). All the mental arithmetic math problems were automatically displayed on the computer screen one by one. The whole test was conducted from 7 p.m. to 9 p.m. EEG data were recorded in a sound-attenuated and light-, temperature-, and humidity-controlled room.

### 2.3. EEG Data Preprocessing

EEG data from only 18 subjects were analysed, because the other two were excluded in the present analysis owing to the big head movements when recording EEG data. Ten pieces of five seconds of artifact-free continuous EEG data (containing no eye blinks, slow eye movements, electrocardiogram artifacts (eliminated by FastICA), and no baseline drift (removed by baseline correction)) were chosen from each condition by EEGLAB. These pieces of data were then downsampled from 1000 Hz to 256 Hz. After digital FFT filtering to extract the EEG basic rhythms (*δ*: 2-4 Hz, *θ*: 4-8 Hz, *α*1: 8-10 Hz, *α*2: 10-13 Hz, and *β*: 13-30 Hz), the MI (see [9] for detailed definition and description) between all pairs of EEG channels was computed by a software written by Moddemeijer [25], obtaining an undirected 19 × 19 AM.

### 2.4. Mental Fatigue Evaluation

In this study, a universal EEG power algorithm, (*θ* + *α*1 + *α*2)/*β* [22–24], was computed to estimate the level of mental fatigue. Nine EEG channels, including F3, C3, P3, F4, C4, P4, Fz, Cz, and Pz (distributed in the middle of the brain), were selected for mental fatigue evaluation based on our existing study [11]. The ratio of (*θ* + *α*1 + *α*2)/*β* is calculated on the average of these 9 EEG channels.

### 2.5. Computation of the Network Characters

**Figure 2:**
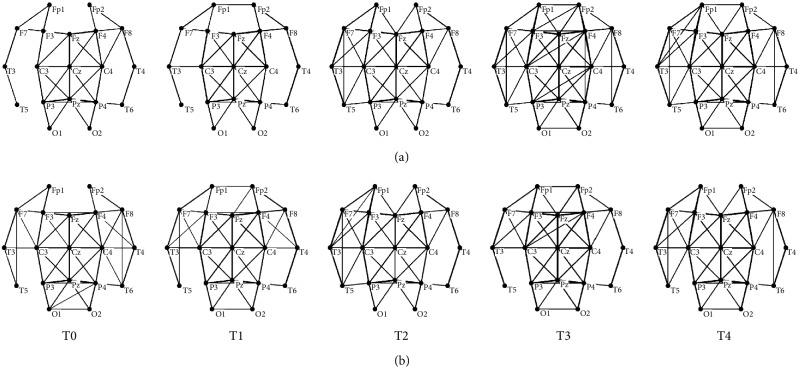
The brain functional networks during the mental fatigue process constructed with two methods. Networks were drawn by Pajek: (a) a threshold of *T* = 0.35; (b) a fixed degree of *K* = 5.

**Figure 3:**
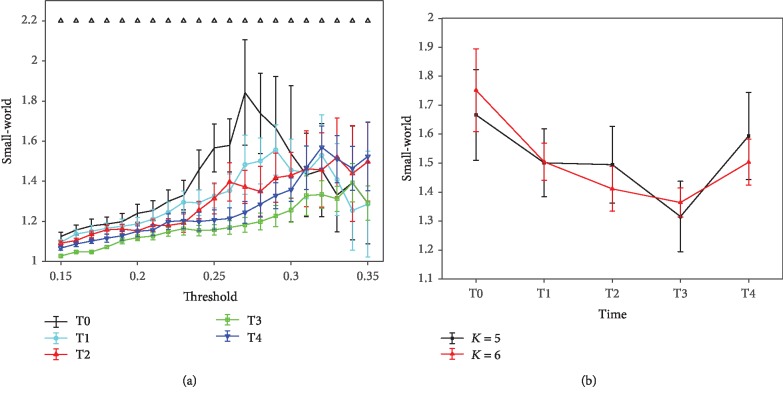
Mean *small-world* of the *α*1 rhythm at the task state with these two different methods. 250 random networks (keep both the number of nodes and the degree distribution constant) were generated based on the brain functional networks for the *small-world* calculation. Each plot shows the mean and standard deviation of all subjects. (a) Results obtained with a series of thresholds (0.15 ≤ *T* ≤ 0.35, with increments of 0.01). Upper triangle refers to where the statistical difference between the five groups is significant (ANOVA, *p* < 0.001). (b) Results obtained with two different degrees *K* = 5 and 6. Results of ANOVA analysis are given in Table 2.

In this part, only binary AM was considered in network construction. AM is a means of representing that the nodes in a network are adjacent to the other nodes. To obtain the binary AM, the MI values were set to zero when the MI value was smaller than a specific threshold; otherwise, to 1. There are two widely used methods to determine the threshold for brain functional network construction. One way is to use the weight (MI) of the functional connectivity as the threshold (Figure 2(a) is an example) [17, 18]. This method can sufficiently reflect the weight information of the functional connectivities. That is, if the whole weights in an AM are higher than that in another one, the corresponding brain functional network would contain more edges, directly resulting in higher *C* and shorter *L*. Therefore, the differences between these two kinds of brain functional networks can be markedly distinguished. The other method is to fix the degree constant in the network (Figure 2(b) is an example) [12]. The purpose to keep the degree fixed is to compare the topological structures of the networks without bias from differences in mean weights. By fixing the degree, all the networks have the same number of nodes and edges; the only differences are in the spatial arrangement [21]. The determination of the threshold in these two methods followed two general principles. Firstly, ensure no isolated nodes in the network. As shown in Figure 2(a), when the threshold is over 0.35, the network of T0 would have isolated nodes. Secondly, the results of the network features are enlarged among T0, T1, T2, T3, and T4. As shown in Figure 3(a), the difference of *small-world* feature among T0, T1, T2, T3, and T4 is greater when the thresholds are between 0.25 and 0.3, which the corresponding average degree is about *K* = 5 or 6 (*K* is the average number of edges per node). Therefore, a series of thresholds between 0.15 and 0.35 with increments of 0.01 and a fixed degree *K* = 5 or 6 were applied to calculate the network characters (*C*, *L*, and *small-world*) in these two methods.

*C* and *L* were defined and described in Equations (1) and (2), respectively [16], where *N* is the set of all nodes in the network, *n* is the total number of nodes, and (*i*, *j*) is the link between node *i* and node *j* (*i*, *j* ∈ *N*). In Equation (1), *C*_*i*_ is a ratio of the actual number of links between the neighbours of node *i* to the total possible number of links between the neighbours of node *i*; *K*_*i*_ represents the number of all neighbour nodes adjacent to node *i*, then at most *K*_*i*_(*K*_*i*_‐1)/2 links exist between them (this occurs when the network has no isolated nodes); and *E*_*i*_ means the actual number of links between the neighbour nodes. In Equation (2), *l*_ij_ is the shortest path length between node *i* and node *j*. A fast algorithm to compute *C* was given by Alon et al. [26]. And the shortest path length was calculated by Dijkstra algorithm [27]:

(1) $$\begin{array}{c} C=\frac{1}{n}\underset{i\in N}{\sum }C_{i},C_{i}=\frac{2E_{i}}{K_{i}\left(K_{i}-1\right)}, \end{array}$$

(2) $$\begin{array}{c} L=\frac{1}{n\left(n-1\right)}\underset{i\neq j\in N}{\sum }L_{ij}. \end{array}$$

The *small-world* characteristic of a network is firstly proposed by Micheloyannis et al. [17] and measured by *C* ≫ *C*_rand_ and *L* ≥ *L*_rand_ based on a random network. *C*_rand_ and *L*_rand_ are *C* and *L* of the random network corresponding to the brain functional network. In this study, we used the ratio of (*C*/*C*_rand_)/(*L*/*L*_rand_) > 1, recommended by Humphries et al. [28], to estimate the *small-world* character. The random network was generated from the experimentally obtained network by a constrained shuffle of the edges among nodes, keeping both the number of nodes and the degree distribution constant. The random networks were generated with a procedure described by Maslov and Sneppen [29].

Before calculating network parameters, one-way analysis of variance (ANOVA) was performed on the mean MI of the original full-weighted AM between 5 time points for every EEG rhythm at the resting state and task state to identify the valid dataset for brain functional network analysis. Only the EEG rhythm with significant statistical difference (*p* < 0.05) would be considered for further network analysis.

### 2.6. Statistical Analysis

One-way ANOVA was implemented to identify significant statistical differences between the 5 time points (T0, T1, T2, T3, and T4). This ANOVA analysis was carried out for the algorithm of (*θ* + *α*1 + *α*2)/*β*, the mean MI of *δ*, *θ*, *α*1, *α*2, and *β* rhythms, as well as the graph parameters (*C*, *L*, and *small-world*). Results are demonstrated as the mean ± standard deviation. A significant level is reported at *p* < 0.05.

## 3. Results

**Figure 4:**
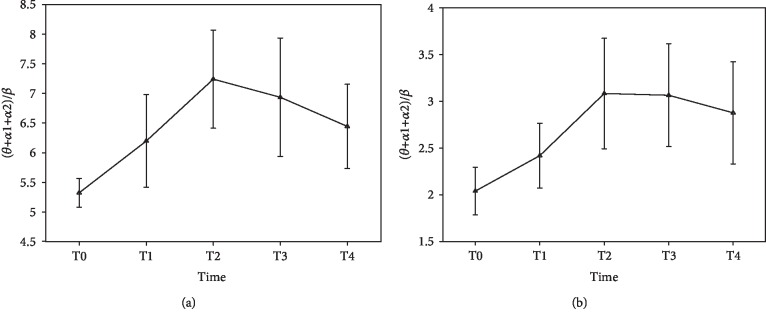
The mean spectral power ratio of (*θ* + *α*1 + *α*2)/*β* plotted over time: (a) results of resting state; (b) results of task state.

Figure 4 shows the results of the ratio of (*θ* + *α*1 + *α*2)/*β* plotted over time. Both in the resting state (Figure 4(a)) and task state (Figure 4(b)), the ratio significantly increases before T2 (ANOVA, *p* < 0.05; see Table 1) and has a small decrease at T3 and T4. But no significant statistical differences are observed in these decreases (ANOVA, *p* > 0.05; see Table 1).

**Figure 5:**
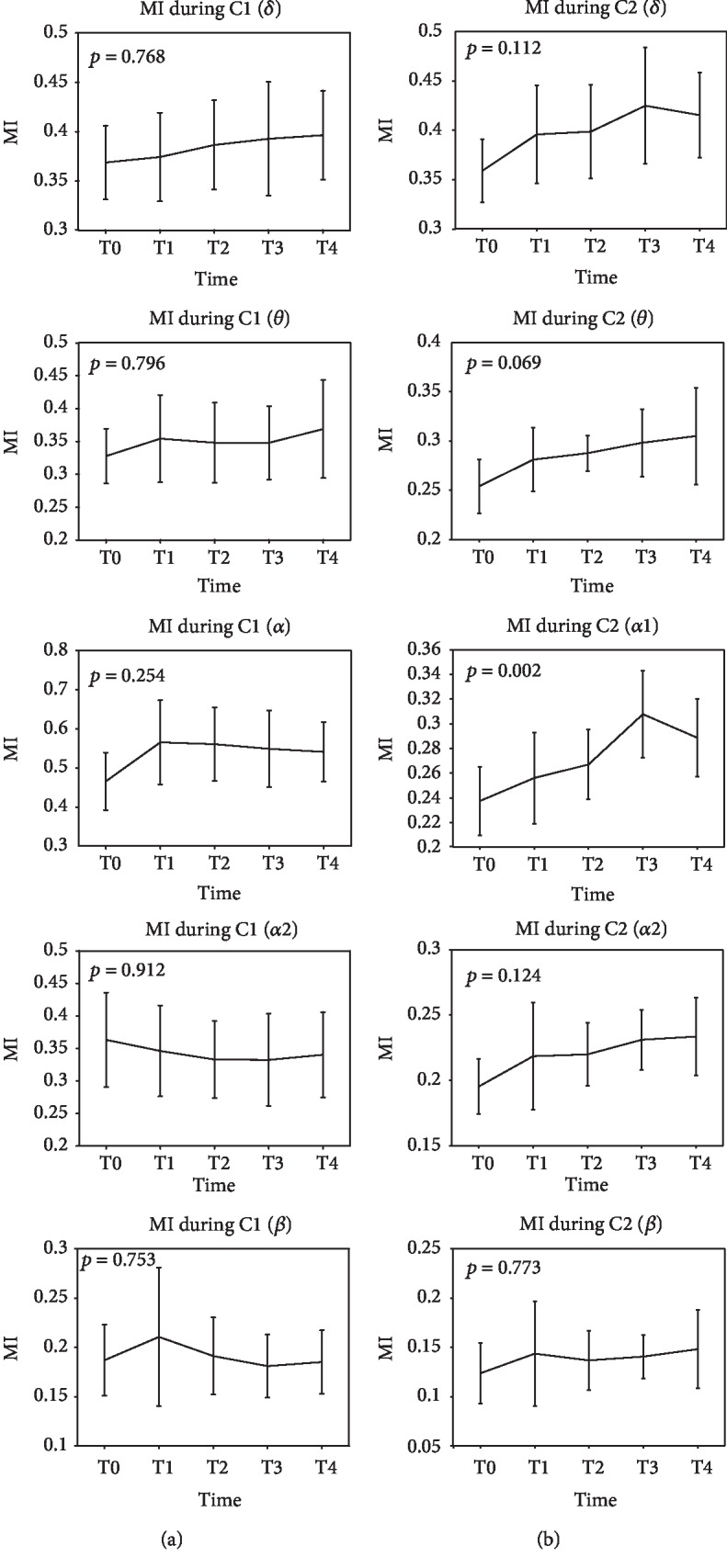
Mean MI (mutual information) of the *δ*, *θ*, *α*1, *α*2, and *β* rhythms during T0, T1, T2, T3, and T4. Firstly, average all adjacency matrices for each subjects. And then, compute the mean MI for all subjects. Figures of column (a) are the results of C1 (resting state); figures of column (b) are the results of C2 (task state).

Results of the mean MI for every EEG rhythm are given in Figure 5. Significant difference (ANOVA, *p* = 0.002) is observed only in *α*1 (alpha1) rhythm at the task state among T0, T1, T2, T3, and T4, which shows that the mean MI increases before T3 and has a small decrease at T4. There are no significant statistical differences in another nine group data (ANOVA, *p* > 0.05). Therefore, just the EEG data from *α*1 rhythm during the task state are considered for further analysis.

**Figure 6:**
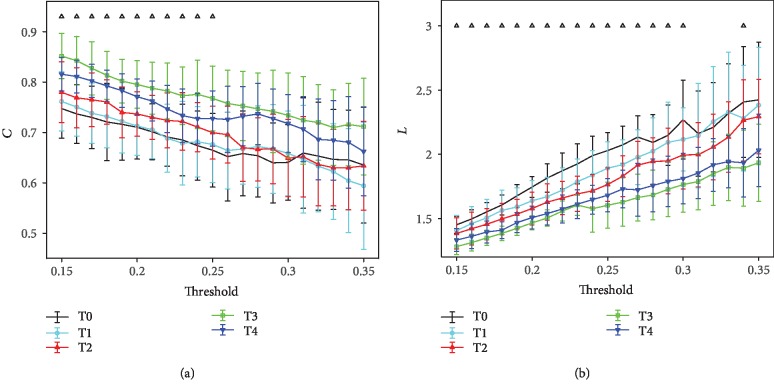
Mean network characters of the *α*1 rhythm at the task state with different values of thresholds (0.15 ≤ *T* ≤ 0.35, with increments of 0.01). The bars indicate the standard deviation of the mean. Upper triangle refers to where the statistical difference between the five groups is significant (ANOVA, *p* < 0.05): (a) results of mean *C*; (b) results of mean *L*.

In Figure 6, it shows the results of mean *C* and *L* for *α*1 rhythm at the task state with a series of thresholds (0.15 ≤ *T* ≤ 0.35, with increments of 0.01). As depicted in Figure 6, a significant increase in *C* (0.15 ≤ *T* ≤ 0.25; ANOVA, *p* < 0.05) and decrease in *L* (0.15 ≤ *T* ≤ 0.3; ANOVA, *p* < 0.05) can be observed at successive stages before T3, and a small irregularity is presented at T4. No significant statistical differences are obtained at 0.26 ≤ *T* ≤ 0.35 (ANOVA, *p* > 0.05) for *C* and at 0.31 ≤ *T* ≤ 0.35 (ANOVA, *p* > 0.05) for *L*.

Figure 3(a) shows the results of *small-world* for *α*1 rhythm at the task state. The *small-world* characteristic significantly decreases over time before T3 for threshold values lower than 0.30 (ANOVA, *p* < 0.001), and a small increase exists at T4. No regular changes are showed when 0.31 ≤ *T* ≤ 0.35. To control the influences of mean MI between the groups, additional results are obtained with the fixed degree of *K* = 5 and 6. No regular variation trend and statistical differences are observed for *C* and *L* (not shown in this paper). But the changing pattern of *small-world* (Figure 3(b)) is in line with that in Figure 3(a). The *small-world* value has a reduction before T3 when the degree is fixed at *K* = 5 and 6 (ANOVA, *p* < 0.01, except between T1 and T2 at *K* = 5; see Table 2) and a large rise at T4 (ANOVA, *p* < 0.01; see Table 2).

## 4. Discussion

In the present study, we attempted to study the effects of mental fatigue on the *small-world* brain functional network organization. For the purpose of confirming the levels of mental fatigue after completing the 25-minute task of mental arithmetic math problems, we chose a widely acceptable algorithm of (*θ* + *α*1 + *α*2)/*β* to estimate mental fatigue [22–24]. Eoh et al. [22] and Jap et al. [23] believed that the algorithm of (*θ* + *α*1 + *α*2)/*β* was a very reliable mental fatigue indicator since it clearly indicated the increasing mental fatigue as the ratio between the slow wave and fast wave activities increased. Sauvet et al. [24] have proved the ratio of (*θ* + *α*1 + *α*2)/*β* as the best index for mental fatigue estimation compared with *θ*_rel_, *α*_rel_, *β*_rel_ (the relative power for each rhythm calculated as a ratio of the total EEG spectral power), and fuzzy logic fusion (*α*, *β*). Previous researchers have found increases in *θ* and *α* activities and a decrease in *β* activity during mental fatigue [22, 23]. But in this part, *δ* activity was excluded and not investigated in the algorithm, because it is the reflection of the sleeping state, overlaps with the artifacts, and is not expected to demonstrate high activity during these tasks [22, 24]. In brief, (*θ* + *α*1 + *α*2)/*β* ratio is a reasonable and reliable index in mental fatigue detection. The results of this ratio plotted over time in this study suggested that mental fatigue can be induced by continuous mental arithmetic tasks.

ANOVA analysis was performed on mean MI of the original full-weighted AM to identify the valid dataset for brain functional network analysis. It is generally known that the network structure can be completely determined by the associated AM. The MI, as the basic elements of the AM, has been proved to be a valid approach to identify the eyes-closed state and eyes-open state [9]. Similar applications of mean MI were also found in other studies [30–32]. Therefore, basing on the significance of the mean MI in demonstrating network features, we statistically analysed the mean MI at all rhythms during the two states. The results showed that significant differences of the mean MI among T0, T1, T2, T3, and T4 were gained only in *α*1 (8-10 Hz) rhythm during the task state (see Figure 5), which can reveal that *α*1 is the most sensitive rhythm in response to mental fatigue. Sun et al. [13] also extracted the same EEG rhythm in functional cortical connectivity analysis of mental fatigue. Thus, only *α*1 rhythm at the task state was considered for brain functional network construction.

The variation tendency of MI had a little inconsistency with (*θ* + *α*1 + *α*2)/*β* ratio. The MI dropped at T3, whereas the (*θ* + *α*1 + *α*2)/*β* ratio decreased at T2. But no significant statistical differences were observed between T2 and T3 for the ratio. Therefore, the mean MI can also be applied to evaluate mental fatigue. Besides, both of these two indicators are nonmonotonically increasing at the middle or end of the test time. This similar changing regularity in mental fatigue detection was also obtained in previous studies [13, 23]. Moreover, consistent variation tendency was obtained between MI and network characters (*C* and *L*). With the increasing of mean MI, *C* increased and *L* decreased. In the controlled tests inducing fatigue with sleep deprivation [15], it reported that the results of *C* and *L* show highly coincident with ours.

In fact, the results of the MI and network characters can explain the neural mechanisms of mental fatigue. We can infer from the increase of MI that with the increasing mental fatigue, the network was expected to have more edges for a given value of *T*. More network edges in a network can naturally result in a higher *C* and shorter *L*, which implies a more efficient transfer of information among cortical nodes. In other words, there were more functional connectivities activated on the basis of prior brain functional networks (see Figure 2(a)), in line with the results reported by other researchers [13–15]. Previous studies have also pointed out that more functional brain areas would be activated for keeping successful performances in the continuing attention task [33, 34], which demonstrated a good consistency in our results of the increasing number of functional connectivities. Moreover, the increase of MI or functional connectivities during the mental fatigue forming process can reveal the better synchronization in *α*1 among the different brain areas [15, 35, 36]. In conclusion, the brain should activate more functional connectivities to complete the same task at the mental fatigue stage, resulting in the further aggravation of mental fatigue.

Besides, the increasing regularity of functional connectivities was in line with the neural efficiency hypothesis. According to neural efficiency hypothesis, more intelligent individuals require less brain activations to fulfill a task, and easier tasks are inclined to produce lower brain activations compared with difficult tasks [18, 37–40]. At the mental fatigue stage, the ability to deal with problems decreased, which is like lower intelligent individuals. In order to accomplish the same task, a person at the mental fatigue stage and a person with lower intelligence had similar reflections of brain activations that more functional brain regions were activated.

A graph encapsulating the *small-world* network structure was suggested be optimal for synchronizing neural activities between different brain regions [41–44]. And it was diagnosed by high *C* and short *L*. At the mental fatigue stage, *C* was significantly higher and *L* was evidently shorter. These principal findings indicated that the brain functional network modulated the neuronal organization to be more efficient for information transmission and processing. Thus, the brain can perform well in demanding sustained tasks at the fatigue stage. Furthermore, with the deepening mental fatigue, the changes in functional connectivities displayed a loss of *small-world* features computed as a function of the threshold (0.15 ≤ *T* ≤ 0.35). To eliminate the influences of discrepancies in mean MI and keep the numbers of edges equal in graphs, we repeated the analysis by calculating *small-world* with a constant degree of *K* = 5 and 6 [17, 18]. These two analysis results were in agreement with each other (see Figure 3). The decrease of *small-world* uncovered that the optimal brain functional network structure was slowly destroyed by the increasing level of mental fatigue. Previous researchers have reported that the changes in brain dysfunctions indicated a loss of *small-world* properties, such as in schizophrenia [17], in Alzheimer's disease [21], and in children with attention-deficit/hyperactivity disorder [45], which implied an agreement with the results of mental fatigue in the reduction of *small-world*. Even if the *small-world* property weakened at the mental fatigue stage, the brain still maintained *small-world* network organizations because the values were all over than 1 [28]. And the *small-world* brain functional network structure recovered at T4 when participants realized that the experiment was coming to an end (relaxing their attention). Moreover, the loss of *small-world* features seemed to be tuned during sleep. Ferri et al. [12] and Dimitriadis et al. [46] noted that the *small-world* feature of the brain functional network was strengthened at the sleep stage. Koenis et al. [47] also stressed that sleep-related processes played an important role in the maintenance of an optimal *small-world* topology of the brain functional network. These findings indicated that sleep can recover the *small-world* network properties of the brain because of its disorganizations during daytime. Besides, the brain functional networks showed a shift toward more random network structures in *α*1 rhythm reflected by a decrease in *small-world*.

Our current study still has a limitation. Using scalp EEG signals has some disadvantages, for instance, the coarse spatial resolution of scalp EEG. If more EEG channels (32, 64, or 128) were included, the results may be more reliable, as well as more findings may be uncovered.

## 5. Conclusions

In the present study, a group of strictly controlled experiments were conducted to explore the effects of mental fatigue on the *small-world* brain functional network organization. To this end, mental fatigue was induced by the mental arithmetic math task and then validated by the algorithm of (*θ* + *α*1 + *α*2)/*β*. The increased ratio of (*θ* + *α*1 + *α*2)/*β* suggested that the included individuals were induced to mental fatigue. For the explorations of mental fatigue with complex network theories, we performed ANOVA analysis on the mean MI between 5 time points for every rhythm to identify the valid dataset, and only the data of *α*1 (8-10 Hz) rhythm during the task state were emerged. The results of graph analysis showed that *C* increased and *L* and *small-world* feature decreased with the deepening mental fatigue. Our present findings indicated that more functional connectivities were activated at the mental fatigue stage for efficient information transmission and processing, resulting in a loss of *small-world* features.

## Figures and Tables

**Table 1:** ANOVA results (*p* values) of the spectral power ratio ((*θ* + *α*1 + *α*2)/*β*) for the resting state and task state between different times. Table 1 is corresponding to Figure 4.

State	T0-T1	T1-T2	T2-T3	T3-T4
Resting state	0.036	0.044	0.591	0.369
Task state	0.048	0.032	0.962	0.621

**Table 2:** ANOVA results (*p* values) of *small-world* for *K* = 5 and 6 between different times. Table 2 is corresponding to Figure 3(b).

*K*	T0-T1	T1-T2	T2-T3	T3-T4
5	4.3*e*‐12	0.78	5.0*e*‐15	4.0*e*‐28
6	9.8*e*‐30	2.68*e*‐09	0.001	4.8*e*‐19

## Data Availability

The data used to support the findings of this study are available from the corresponding authors upon request.

## Abbreviations

EEG: Electroencephalogram
MI: Mutual information
*C*: Clustering coefficient
*L*: Characteristic path length
ANOVA: Analysis of variance
EEG DAQ: EEG data acquisition
AM: Adjacency matrix
p.m.: Post meridiem.

## References

[1] Boksem M. A. S., Tops M. (2008). Mental fatigue: costs and benefits. *Brain Research Reviews*.

[2] Chaudhuri A., Behan P. O. (2004). Fatigue in neurological disorders. *The Lancet*.

[3] Lal S. K. L., Craig A. (2001). A critical review of the psychophysiology of driver fatigue. *Biological Psychology*.

[4] Lal S. K. L., Craig A. (2002). Driver fatigue: electroencephalography and psychological assessment. *Psychophysiology*.

[5] Lal S. K. L., Craig A., Boord P., Kirkup L., Nguyen H. (2003). Development of an algorithm for an EEG-based driver fatigue countermeasure. *Journal of Safety Research*.

[6] Lee L., Harrison L. M., Mechelli A. (2003). The functional brain connectivity workshop: report and commentary. *Network*.

[7] Liu P., Shen H., Ji S. (2019). Functional connectivity pattern analysis underlying neural oscillation synchronization during deception. *Neural Plasticity*.

[8] Nguyen T., Potter T., Nguyen T., Karmonik C., Grossman R., Zhang Y. (2016). EEG source imaging guided by spatiotemporal specific fMRI: toward an understanding of dynamic cognitive processes. *Neural Plasticity*.

[9] Jin S. H., Jeong W., Lee D. S., Jeon B. S., Chung C. K. (2014). Preserved high-centrality hubs but efficient network reorganization during eyes-open state compared with eyes-closed resting state: an MEG study. *Journal of Neurophysiology*.

[10] Li G., Li B., Jiang Y., Jiao W., Lan H., Zhu C. (2018). A new method for automatically modelling brain functional networks. *Biomedical Signal Processing and Control*.

[11] Li G., Li B., Wang G., Zhang J., Wang J. (2017). A new method for human mental fatigue detection with several EEG channels. *Journal of Medical and Biological Engineering*.

[12] Ferri R., Rundo F., Bruni O., Terzano M. G., Stam C. J. (2007). Small-world network organization of functional connectivity of EEG slow-wave activity during sleep. *Clinical Neurophysiology*.

[13] Sun Y., Lim J., Meng J., Kwok K., Thakor N., Bezerianos A. (2014). Discriminative analysis of brain functional connectivity patterns for mental fatigue classification. *Annals of Biomedical Engineering*.

[14] Lorist M. M., Bezdan E., ten Caat M., Span M. M., Roerdink J. B. T. M., Maurits N. M. (2009). The influence of mental fatigue and motivation on neural network dynamics; an EEG coherence study. *Brain Research*.

[15] Kar S., Routray A. (2013). Effect of sleep deprivation on functional connectivity of EEG channels. *IEEE Transactions on Systems, Man, and Cybernetics: Systems*.

[16] Watts D. J., Strogatz S. H. (1998). Collective dynamics of ‘small-world’ networks. *Nature*.

[17] Micheloyannis S., Pachou E., Stam C. J. (2006). Small-world networks and disturbed functional connectivity in schizophrenia. *Schizophrenia Research*.

[18] Micheloyannis S., Pachou E., Stam C. J., Vourkas M., Erimaki S., Tsirka V. (2006). Using graph theoretical analysis of multi channel EEG to evaluate the neural efficiency hypothesis. *Neuroscience Letters*.

[19] He Y., Chen Z. J., Evans A. C. (2007). Small-world anatomical networks in the human brain revealed by cortical thickness from MRI. *Cerebral Cortex*.

[20] Jin S. H., Lin P., Hallett M. (2011). Abnormal reorganization of functional cortical small-world networks in focal hand dystonia. *PLoS One*.

[21] Stam C. J., Jones B. F., Nolte G., Breakspear M., Scheltens P. (2007). Small-world networks and functional connectivity in Alzheimer’s disease. *Cerebral Cortex*.

[22] Eoh H. J., Chung M. K., Kim S.-H. (2005). Electroencephalographic study of drowsiness in simulated driving with sleep deprivation. *International Journal of Industrial Ergonomics*.

[23] Jap B. T., Lal S., Fischer P., Bekiaris E. (2009). Using EEG spectral components to assess algorithms for detecting fatigue. *Expert Systems with Applications*.

[24] Sauvet F., Bougard C., Coroenne M. (2014). In-flight automatic detection of vigilance states using a single EEG channel. *IEEE Transactions on Biomedical Engineering*.

[25] Moddemeijer R. (1999). A statistic to estimate the variance of the histogram-based mutual information estimator based on dependent pairs of observations. *Signal Processing*.

[26] Alon N., Yuster R., Zwick U. (1997). Finding and counting given length cycles. *Algorithmica*.

[27] Dijkstra E. W. (1959). A note on two problems in connexion with graphs. *Numerische Mathematik*.

[28] Humphries M. D., Gurney K., Prescott T. J. (2006). The brainstem reticular formation is a small-world, not scale-free, network. *Proceedings of the Royal Society B-Biological Sciences*.

[29] Maslov S., Sneppen K. (2002). Specificity and stability in topology of protein networks. *Science*.

[30] Jeong J., Gore J. C., Peterson B. S. (2001). Mutual information analysis of the EEG in patients with Alzheimer’s disease. *Clinical Neurophysiology*.

[31] Jin S. H., Lin P., Hallett M. (2012). Reorganization of brain functional small-world networks during finger movements. *Human Brain Mapping*.

[32] Na S. H., Jin S. H., Kim S. Y., Ham B. J. (2002). EEG in schizophrenic patients: mutual information analysis. *Clinical Neurophysiology*.

[33] Demeter E., Hernandez-Garcia L., Sarter M., Lustig C. (2011). Challenges to attention: a continuous arterial spin labeling (ASL) study of the effects of distraction on sustained attention. *NeuroImage*.

[34] Lawrence N. S., Ross T. J., Hoffmann R., Garavan H., Stein E. A. (2003). Multiple neuronal networks mediate sustained attention. *Journal of Cognitive Neuroscience*.

[35] Boksem M. A. S., Meijman T. F., Lorist M. M. (2005). Effects of mental fatigue on attention: an ERP study. *Cognitive Brain Research*.

[36] Klimesch W. (1999). EEG alpha and theta oscillations reflect cognitive and memory performance: a review and analysis. *Brain Research Reviews*.

[37] Thatcher R. W., Palmero-Soler E., North D. M., Biver C. J. (2016). Intelligence and EEG measures of information flow: efficiency and homeostatic neuroplasticity. *Scientific Reports*.

[38] Grabner R. H., Fink A., Stipacek A., Neuper C., Neubauer A. C. (2004). Intelligence and working memory systems: evidence of neural efficiency in alpha band ERD. *Cognitive Brain Research*.

[39] Neubauer A. C., Grabner R. H., Fink A., Neuper C. (2005). Intelligence and neural efficiency: further evidence of the influence of task content and sex on the brain-IQ relationship. *Cognitive Brain Research*.

[40] Nussbaumer D., Grabner R. H., Stern E. (2015). Neural efficiency in working memory tasks: the impact of task demand. *Intelligence*.

[41] Barahona M., Pecora L. M. (2002). Synchronization in small-world systems. *Physical Review Letters*.

[42] Lago-Fernandez L. F., Huerta R., Corbacho F., Siguenza J. A. (2000). Fast response and temporal coherent oscillations in small-world networks. *Physical Review Letters*.

[43] Latora V., Marchiori M. (2001). Efficient behavior of small-world networks. *Physical Review Letters*.

[44] Masuda N., Aihara K. (2004). Global and local synchrony of coupled neurons in small-world networks. *Biological Cybernetics*.

[45] Liu T., Chen Y., Lin P., Wang J. (2015). Small-world brain functional networks in children with attention-deficit/hyperactivity disorder revealed by EEG synchrony. *Clinical EEG and Neuroscience*.

[46] Dimitriadis S. I., Laskaris N. A., Del Rio-Portilla Y., Koudounis G. C. (2009). Characterizing dynamic functional connectivity across sleep stages from EEG. *Brain Topography*.

[47] Koenis M. M. G., Romeijn N., Piantoni G. (2013). Does sleep restore the topology of functional brain networks?. *Human Brain Mapping*.

